# A 3-day step taper enhances explosive power, repeated sprint, and small-sided game performance in soccer

**DOI:** 10.3389/fspor.2026.1811250

**Published:** 2026-05-13

**Authors:** Mohamed Saifeddin Fessi, Mohamed Elloumi, Mohamed Amine Bouzid, Mehdi Ben Brahim, Wassim Moalla

**Affiliations:** 1 Research Laboratory Sports Performance Optimization (LR09SEP01), National Center of Medicine and Science in Sports (CNMSS), Tunis, Tunisia; 2 Sport Sciences and Diagnostics Research Group, GS-HPE Department, Prince Sultan University, Riyadh, Saudi Arabia; 3 LR 19JS01 EM2S, Education, Motricity, Sport and Health, High Institute of Sport and Physical Education, University of Sfax, Sfax, Tunisia

**Keywords:** microcycle, professional players, repeated sprint ability, taper, training load

## Abstract

**Aims:**

This study aimed to investigate the effects of a three-day step taper on lower-limb explosive power, repeated-sprint performance, and physical and physiological parameters during sport-specific exercises in professional soccer players.

**Methods:**

The study involved 26 outfield professional soccer players from the same team (age: 28.7±4.3 years; body mass: 71.8±7.2 kg; height: 173.7±4.3 cm). Following a 4-week mesocycle of progressive training load, players were randomly assigned to a taper group (TG; *n* = 13), which underwent a three-day step taper with a 50% reduction in training duration while maintaining intensity, and a control group (CG; n = 13), which maintained their routine. Both groups performed two Countermovement jumps with arm swing (CMJAS), a 5×30-m repeated-sprint test, and a Small-Sided Game (SSG) before and after the step tapering. Training load was monitored using the session rating of perceived exertion (sRPE) method.

**Results:**

The results indicated that the TG improved CMJAS height (large effect; d = 1.5; *p* < 0.01) and decreased peak and total time during repeated sprints (small effect; *d* = 0.3; *p* < 0.05). It also enhanced the total covered distance (small effect; *d* = 0.3; *p* < 0.05), as well as accelerations and decelerations during the SSG (moderate effect; *d* = 0.8 and *d* = 0.6, respectively; *p* < 0.05). In contrast, the CG showed significant decreases in CMJAS height (moderate effect; *d* = 1; *p* < 0.01) and increases total sprint times (small effect; *d* = 0.4; *p* < 0.05), indicating reduced performance, with no significant changes in SSG metrics.

**Conclusions:**

The study indicates that a 3-day step taper with a 50% reduction in training duration, while keeping intensity and frequency, effectively improves repeated-sprint ability and explosive power in professional soccer players, without performance loss from prior high training loads.

## 1 Introduction

Sustained high training loads during the season often cause fatigue and reduced performance in professional soccer players (1–3). Therefore, coaches must adopt effective recovery strategies and reduce training loads at the end of each mesocycle (2). Tapering aims to progressively decrease both the physiological and psychological stress of training to optimize performance following intense training (4–6). Previous studies on tapering strategies report wide variations in duration, intensity, volume, and frequency (3–5).

Training load can be reduced through linear, exponential, or step-based procedures (4, 5). Linear tapering involves a continuous and steady reduction of training load, providing a predictable decrease in accumulated fatigue. Exponential tapering utilizes a non-linear decay constant and can be further categorized into “fast decay”, where the load is reduced more aggressively, or “slow decay”, where the reduction is more gradual. Research suggests that exponential fast-decay models are often superior for maximizing physiological adaptations by rapidly reducing fatigue while maintaining training intensity (4, 5). In contrast, step tapering involves a sudden, discrete, and non-progressive reduction in training load (e.g., a fixed 50% drop in volume) maintained throughout the microcycle (6). This model is particularly practical in professional team sports due to its simplicity and ease of implementation within tight competitive schedules.

Previous studies have consistently established the effectiveness of tapering in improving performance in both individual and team sports, regardless of controlled variables such as taper duration, training intensity, volume, and frequency, or the applied tapering approach (5, 6). Indeed, limited evidence on tapering use in soccer has been reported in the sports science's literature (1, 5, 6). Most available studies in soccer have applied a step-taper approach with significant findings (1, 2, 7–9). Fessi et al. (1) found that a 1-week, 25% step reduction in training load while maintaining intensity resulted in a 15% increase in high and very high-intensity game activities. Similarly, Figueiredo et al. (7) identified an improvement in intermittent running performance following an initial 2-week overload, succeeded by a 1-week step reduction of 60% in training volume; training frequency and intensity remained consistent. Likewise, Beltran-Valls et al. (8) demonstrated that a 2-week step reduction in training duration (40%–59%), while maintaining training frequency and intensity, improved muscle power and acceleration, and reduced stress levels. Recently, Krespi et al. (9) reported that a 4-week exponential taper produced superior outcomes in terms of speed, power, and endurance compared to a 4-week linear taper. Most protocols last 1–2 weeks, resulting in insufficient evidence for shorter tapering strategies, especially during congested in-season schedules that include midweek games and tournaments (1, 2, 7). This gap is significant because elite competitions, including the UEFA Champions League knockout stages and domestic cup-league doubles, typically involve 72-hour microcycles where matches are scheduled every 3 days (10). A short, 3-day step taper could be especially beneficial during congested match schedules. Therefore, the present study aimed to assess the impact of a 3-day step taper on lower extremity power, repeated sprint performance, and various physiological and running metrics during sport-specific exercises in professional soccer players.

## 2 Methods

### 2.1 Experimental approach to the problem

**Figure 1:**
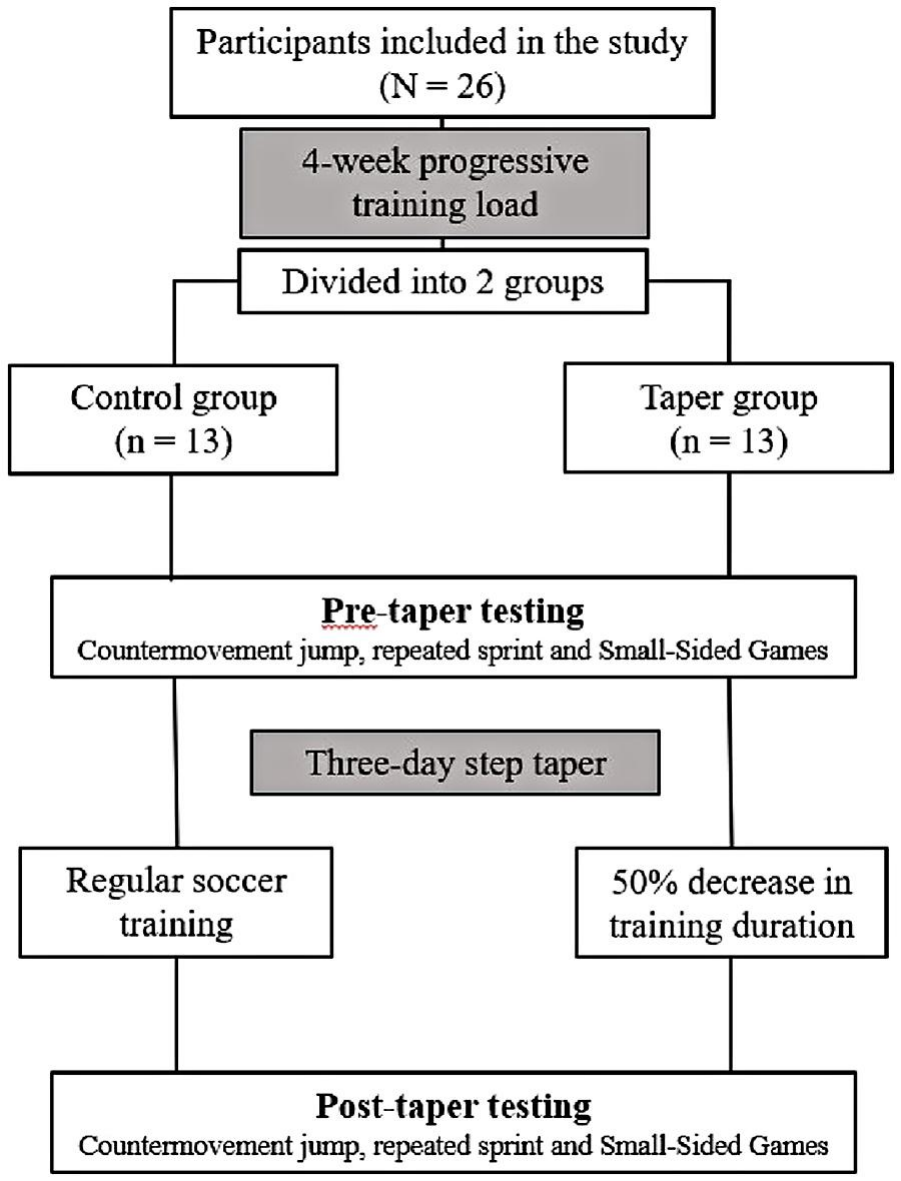
Study procedure overview.

The study implemented a 3-day step taper after a 4-week progressive training mesocycle (Figure 1). The players engaged in five to six training sessions during the pre-taper mesocycle and played an official match weekly. Players completed active recovery training the day after a game, receiving a day off, while those who did not play or played less than 45 min participated in a similar training session. The pre-taper mesocycle training load was adjusted based on the players' previous training records from the preceding mesocycle. Each microcycle included strength and plyometric training sessions once or twice a week, with an increased volume of 10% during the second week. Subsequently, the intensity was individually raised by 3%–5% for the height or distance of vertical and horizontal jumps in plyometric training and increased for strength training weights. Small-Sided Games (SSGs) were utilized as sport-specific training during the experimental weeks. The SSG volume and intensity were increased in alternate weeks. The volume was first adjusted by increasing the duration of each task or the number of tasks performed per session. The intensity was then enhanced by limiting the number of ball touches per player or reducing the playing space (11).

Afterward, during the 4-week mesocycle, all players engaged in active recovery training the day following a game before taking a day off. Subsequently, participants were randomly assigned to either the TG, which underwent the tapering protocol, or the CG, which maintained their regular training routine. The duration of the taper microcycle was set according to previous research (12), which indicates that soccer players experience significant strain due to the demanding professional match schedule, often playing matches with only a 72-hour gap between them. During the 3-day step taper, training session duration was reduced by 50% relative to the pre-taper microcycle, while intensity and frequency were maintained (Table 1). The club's technical/coaching staff designed the training program throughout the 4-week mesocycle and the 3-day step taper.

**Table 1:** The planned training program, rating of perceived exertion, and duration during pre and taper microcycle.

Day	Training program	Planned RPE	Duration (min)
Match day			
Recovery day	Regeneration exercises and low-impact activities	1.5–2	50
Match day +2	Day-off		
Match day −4	Strength training, pressing tasks, and small-sided games	4	90
Match day −3	Activation drills, rondos, and 11 vs. 11	5	90
Match day −2	Preventive strength training, rondos positional games, transition drills, and 11 vs. 11 matches	4	80
Match day −1	Activation drills, small-sided games, and review of tactical keys regarding the match	3	60
Match day			
Match day +1	Regeneration exercises and low-impact activities	1.5–2	50
Match day +2	Day-off		
Pre-taper testing			
Taper day 1	Small-sided games	5	45
Taper day 2	Preventive strength training, rondos positional games, transition drills	4	40
Taper day 3	Small-sided games	3	30
Post-taper testing			

All participants completed the Countermovement jumps with arm swing (CMJAS), to assess lower extremity power, the repeated linear sprint test (5 × 30 m), and participated in 4 vs. 4 with goalkeepers (4 vs. 4 + GK) SSG twice, both pre- and post-taper, separated by a 3-day step taper that included three training sessions. All measurements were conducted in a single session, sequentially following a standard 15-minute warm-up, with a 5-minute rest between each test on the same natural grass soccer field during the last week of April 2023. The temperature was maintained at 23 °C ± 0.9 °C throughout the period. To reduce the impact of diurnal variability on physical performance, testing sessions were held between 10 a.m. and 12 p.m. The study was conducted in accordance with the Declaration of Helsinki and was approved by the Ethics Committee for the Protection of Persons South (E.C.P. SOUTH, Tunisia), approval No. 082/2023, affiliated with the Ministry of Health, Tunisia.

### 2.2 Participants and recruitment

Twenty-six outfield professional soccer players (age: 28.7 ± 4.3 years; body mass: 71.8 ± 7.2 kg; height: 173.7 ± 4.3 cm; professional career: 7.3 ± 3.7 years) from the same team volunteered to participate in the study. All players had regularly trained with the team since the start of the season and consistently participated in the top-level Professional League. Participants were randomly assigned to either the taper group (TG; *n* = 13) or the control group (CG; *n* = 13). The participants included a balanced distribution of playing positions in each group (4 defenders, 5 midfielders, and 4 forwards). To ensure baseline comparability, match exposure was monitored during the 4-week pre-taper period. Players who completed less than 60 min of official match-play underwent standardized compensatory training sessions (top-up) on the same day. This approach ensured that the total daily training load remained consistent across all participants, regardless of individual match duration. *A priori* sample size was calculated according to Faul et al. (13) procedures using the G*Power software (version 3.1.9.4; Kiel University, Kiel, Germany). The *α* and power values were set at 0.05 and 0.85, respectively. Based on the results of Beltran-Valls et al. (8), effect sizes were estimated to be >0.8 (large effect). Twelve participants per group would minimize the risk of committing a type II statistical error. All procedures were completed as standard training observation, and written informed consent was obtained from all the participants.

### 2.3 Training load monitoring

Rating of perceived exertion (RPE) was collected 30 min after each session or match using a modified 10-point Borg scale (14). Data were collected from players by the same fitness coach. Players answered: “How hard was the training session?” Each player completed the scale without the presence of other players and was unable to see the values of other participants to eliminate any influence on their responses. All players were familiarized with these scales, as they are part of their duties during daily training. The training load was then calculated by multiplying RPE by the duration (in minutes) of the session, testing, and/or game (15). The daily training load (a.u.) was monitored throughout the 4-week pre-taper mesocycle and the 3-day step taper to ensure the consistency of the training stimulus between groups. The validity of the RPE-based training load method in soccer has been previously documented (15).

### 2.4 Countermovement jumps with arm swing

The lower extremity power was assessed using a countermovement jump with arm swing (CMJAS) performed with an arm swing (i.e., hands were free to move). Participants were instructed to start by standing with their torso upright. Players performed a rapid downward movement to ∼90° knee flexion, followed by an explosive upward jump with arm swing. The participants completed 3 trials, and the best performance was retained for analysis. Jump height during the CMJAS was measured using the Optojump System (Microgate, Bolzano, Italy).

### 2.5 Repeated linear sprint test

The 5 × 30-m test consisted of five consecutive 30-meter line sprints with 30 s of active, self-paced recovery between each sprint, according to the procedures developed by Castagna et al. (16). Each sprint's duration was measured using an electronic timing system accurate to 0.01 s (Cell Kit Speed-Bow®, Brower Timing Systems, USA), positioned at the start and finish lines, both located 0.5 m above the ground. After each sprint, players decelerated and returned to the start, following a computerized countdown for the next sprint. They were individually tested on natural grass surfaces while wearing soccer boots, with coaches providing standard verbal encouragement throughout the trials. The performance of the repeated sprint test is expressed as follows: The total time represents the sum of the times of five sprints, the peak time denotes the best recorded time, and the sprint decrement (%) is calculated as [100 × (total sprint time/best × 5)] − 100. The ecological validity of the 5 × 30-m sprint test has been previously reported, with the ICC and CV values of 0.96–0.98 and 1.2% for the best and total times, and 0.34 and 66.1% for the sprint decrement (16, 17).

### 2.6 Small-sided games

The 4 vs. 4 + GK SSGs (total of 10 players on the pitch), included free-touch tasks. Two four-minute working intervals were implemented, with 2 min for recovery. Pitch dimensions were 22 m × 32 m (704 m^2^), and the playing areas were approximately 70.4 m^2^ per player, (calculated based on all 10 players present on the pitch). A standard match-size goal was used throughout the interventions. The offside rule was excluded, and the same coaching staff provided standard encouragement in pre- and post-taper sessions. All throw-ins for restarting the game were performed using goalkeepers from their standard positions (18, 19). These SSGs were regularly integrated into the players' training routines. The physical and physiological responses to SSGs were monitored using portable Global Positioning System (GPS) technology with an integrated accelerometer and heart rate (HR) monitoring (GPSports SPI Pro X, Canberra, Australia). GPS devices were placed into a harness between the player's shoulder blades. This device provides raw position, velocity, and distance data at 15 Hz (15 samples per second) and averages every three raw data points to provide a sampling frequency of 5 Hz. GPS systems have been shown to provide valid and reliable estimates of distance and velocity during linear, multidirectional activities in field-based team sports (20). All devices were activated 15 min before data collection and checked to ensure connection to satellites before data collection (20). Players used the same device for both pre- and post-taper SSG sessions to avoid inter-unit errors. Based on the SSG data, total distance, high speed (>19.8 km.h^−1^), sprint distance (>25.5 km.h^−1^), average metabolic power (W·kg^−1^), high metabolic power distance (m; ≥20 W·kg^−1^), accelerations (n), decelerations (n), and average and maximal HR were retained for analysis.

### 2.7 Statistical analysis

Data are expressed as means ± standard deviations (mean ± SD). The data were checked for normality using the Kolmogorov–Smirnov test. A two-way analysis of variance (ANOVA) was performed for time (pre- and post-taper) and group (Taper and control) to assess the interaction of lower extremity power, repeated sprint performance, and various physiological and running metrics during SSG. An independent Student's *t*-test was used to compare the pre-taper measurements, training load, and RPE between the two groups during the 3-day taper. A paired Student's *t*-test compared training load and RPE during the 3-day taper to the equivalent day of the pre-taper microcycle. Comparisons of pre- and post-taper lower extremity power, repeated sprint performance, and various physiological and running metrics during SSG were made using a Student's *t*-test for each group. Effect sizes (Cohen's *d*) were interpreted as trivial (<0.2), small (0.2–0.6), moderate (0.6–1.2), large (1.2–2.0), and very large (>2.0) (21). The data were analyzed using SPSS software version 26, and the significance level was set at *p* ≤ 0.05.

## 3 Results

**Figure 2:**
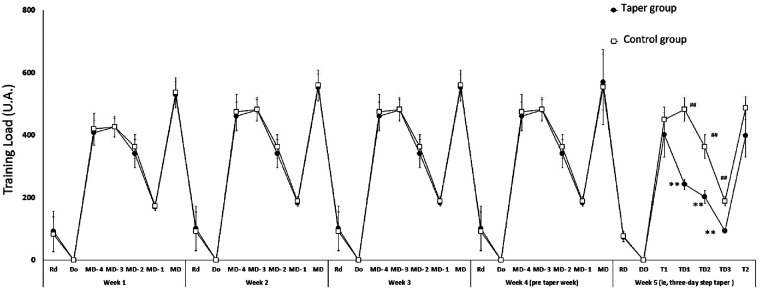
The pattern of training load performed by both groups during a 4-week mesocycle and the 3-day taper. Rd, recovery day; Do, day-off; MD, match day; T, testing session; TD, taper day. **Difference between the 3-day taper and the corresponding day of the pre-taper microcycle at *p* < 0.01. ^##^Difference between two group training loads at *p* < 0.01.

The training load achieved by both groups throughout the experimental period are presented in Figure 2. During the 4-week pre-taper mesocycle, no significant differences were observed between the TG and the CG for mean daily or weekly training loads (*p* > 0.05; Figure 2). The training load achieved by TG during the 3-day step taper was significantly lower than that of the CG (*p* < 0.01; Figure 2), while RPE values showed no difference (*p* > 0.05; Figure 2).

 Table 2 presents lower extremity power, repeated sprint performance, and SSG metrics for both groups before and after tapering. No pre-taper (baseline) differences were found between groups for all measurement variables (*p* > 0.05), except for RPE after SSGs, where a significant difference was observed (TG vs. CG 6.69 ± 1.18 a.u. vs. 7.50 ± 0.61 a.u., *p* < 0.05).

**Table 2:** Countermovement jumps with arm swing, repeated sprint performance and various physiological and running metrics during small-sided games of both groups at pre and post-taper measurements.

	Taper group				Control group			
Variables	Pre taper	Post-taper	Δ%	*d*; Magnitude	Pre taper	Post-taper	Δ%	*d*; Magnitude
Countermovement jump with arm swing (cm)	55.96 ± 2.10	59.22 ± 2.10**	5.8	1.6; *Large*	56.10 ± 2.44	53.44 ± 2.72**	−4.7	1.0; *Moderate*
Repeated linear sprint test								
Peak time (s)	4.34 ± 0.21	4.27 ± 0.21*	−1.6	0.3; *Small*	4.35 ± 0.56	4.40 ± 0.11	1.2	0.1; *Trivial*
Total time (s)	22.47 ± 1.06	22.15 ± 1.06*	−1.4	0.3; *Small*	22.46 ± 1.04	22.83 ± 1.05*	1.6	0.4; *Small*
Sprint decrement (%)	3.35 ± 0.99	3.39 ± 1.36	1.2	0.0; *Trivial*	3.38 ± 0.92	3.43 ± 1.41	1.4	0.0; *Trivial*
Small-sided game (4 vs. 4 with goalkeepers)								
Total distance (m)	1,000.10 ± 251.54	1,020.33 ± 257.31*	2.1	0.3; *Small*	1,012.15 ± 85.87	1,011.98 ± 85.97	0.0	0.0; *Trivial*
High-speed distance (m)	30.68 ± 7.08	33.58 ± 6.82*	9.5	0.4; *Small*	28.35 ± 8.22	28.37 ± 8.17	−0.1	0.0; Trivial
Sprint distance (m)	23.95 ± 4.19	27.37 ± 3.70**	14.3	0.9; *Moderate*	22.91 ± 5.05	22.81 ± 4.89	0.4	0.0; *Trivial*
Average metabolic load (W·kg^−1^)	11.74 ± 0.81	12.22 ± 0.45*	4.1	0.7; *Moderate*	11.30 ± 1.12	11.29 ± 1.14	−0.1	0.0; *Trivial*
High metabolic load distance (m; W·kg^−1^)	220.38 ± 37.51	225.24 ± 29.47*	2.2	0.2; *Small*	205.72 ± 38.23	205.45 ± 38.34	−0.1	0.0; *Trivial*
Accelerations (n)	21.96 ± 4.15	25.46 ± 4.23*	15.9	0.8; *Moderate*	22.24 ± 8.79	22.19 ± 8.95	−0.2	0.0; *Trivial*
Decelerations (n)	22.53 ± 5.87	26.04 ± 5.99*	15.6	0.6; *Moderate*	22.02 ± 7.67	22.11 ± 7.65	−0.4	0.0; *Trivial*
Average heart rate (bpm)	162.28 ± 10.53	157.80 ± 10.19*	−2.8	0.4; *Small*	161.48 ± 19.17	161.37 ± 19.16	0.1	0.0; *Trivial*
Maximal heart rate (bpm)	184.50 ± 5.66	184.19 ± 5.44	−0.2	0.0; *Trivial*	185.46 ± 9.07	185.88 ± 9.10	0.2	0.0; *Trivial*
Rating of perceived exertion (a.u.)	6.69 ± 1.18	6.65 ± 1.14	−0.6	0.0; *Trivial*	7.50 ± 0.61***	8.11 ± 0.17**	8.1	1.4; *Large*

Magnitude of change (*d*) is expressed as: trivial (<0.2), small (0.2–0.6), moderate (0.6–1.2), and large (1.2–2.0).

* Difference between pre-and post-taper data at *p* < 0.05.

** Difference between pre-and post-taper data at *p* < 0.01.

*** Difference between two group pre-taper data at *p* < 0.05.

**Figure 3:**
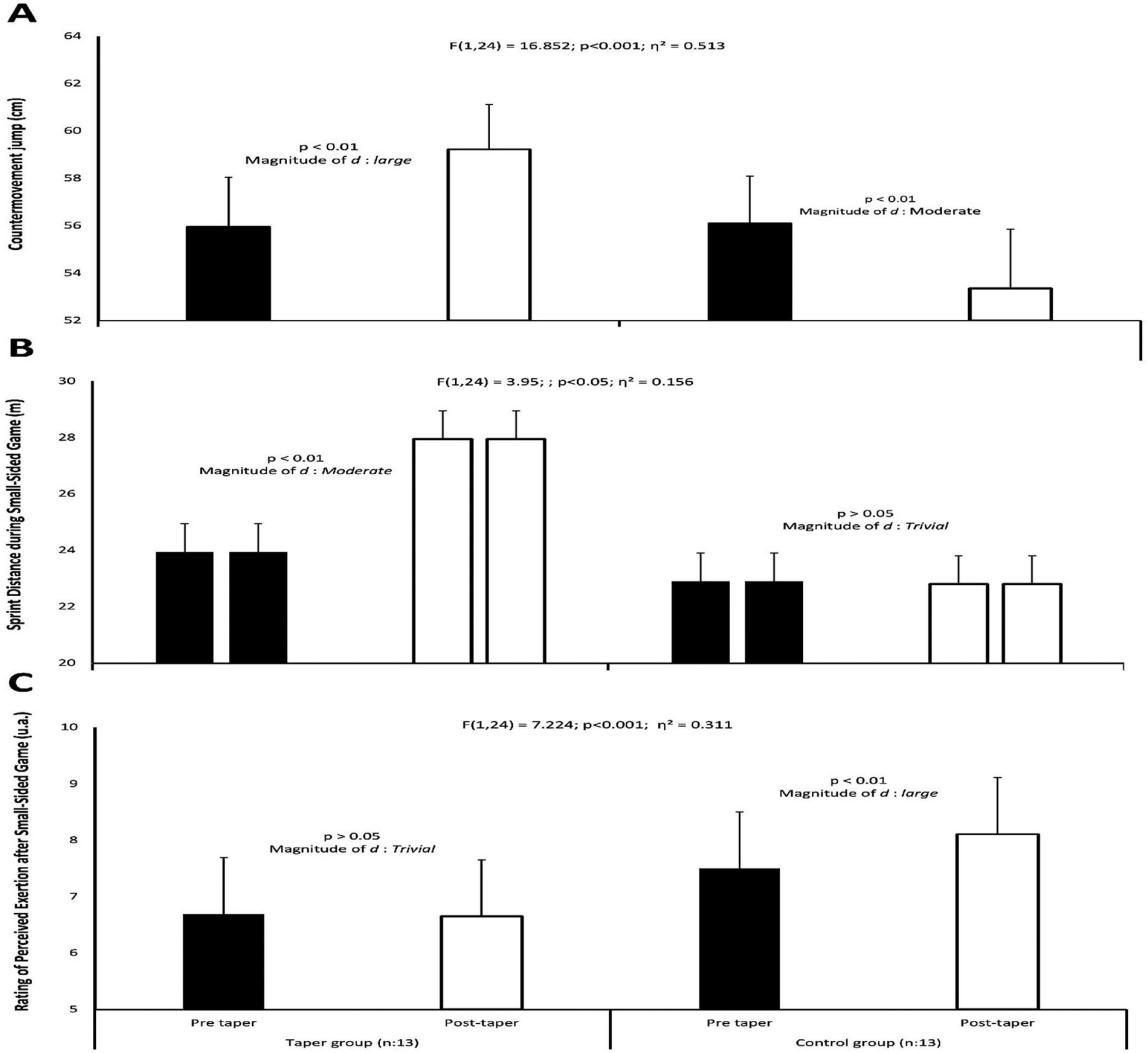
Between-group interaction effects on **(A)** countermovement jump with arm swing performance; **(B)** sprint distance covered during 4 vs. 4 with goalkeepers small-sided games and **(C)** Rating of perceived exertion reported after small-sided games. Magnitude of change (*d*) is expressed as: trivial (<0.2), small (0.2–0.6), moderate (0.6–1.2), and large (1.2–2.0).

The between-groups analysis revealed a significant time × group interaction for the CMJAS [*F*_(1,24)_ = 16.852; *η*^2^ = 0.513; *p* < 0.001; Figure 3A], sprint distance covered during SSGs [*F*_(1,24)_ = 3.95; *η*^2^ = 0.156; *p* < 0.05; Figure 3B], and RPE after SSGs [*F*_(1,24)_ = 7.224; *η*^2^ = 0.311; *p* < 0.001; Figure 3C]. No time × group interaction was observed for the other parameters.

Significant improvements were observed in CMJAS performance in the TG post-taper (5.8%, large effect, *d* = 1.6; *p* < 0.01). Both peak and total times during repeated sprints decreased significantly (−1.6%, *d* = 0.3, and −1.4%, *d* = 0.3, small effect, respectively; *p* < 0.05). However, no significant difference was noted for sprint decrement. The total distance covered during the SSGs significantly increased post-taper (2.0%, small effect, *d* = 0.3; *p* < 0.05). Significant differences were observed for high speed (small effect, *d* = 0.4; *p* < 0.05), sprint distance (moderate effect, *d* = 0.9; *p* < 0.01), and high metabolic load distance (small effect, *d* = 0.2; *p* < 0.05) during the post-taper. Post-taper, the average metabolic load, acceleration, and deceleration demonstrated significant increases (4.1%, *d* = 0.7; 15.9%, *d* = 0.8; and 15.6%, *d* = 0.6, moderate effect, respectively; *p* < 0.05). Average HR post-taper showed a significant decrease (−2.8%, small effect, *d* = 0.4; *p* < 0.05). However, no significant differences were observed for maximal HR and RPE between pre- and post-taper measurements in the TG.

In the CG, CMJAS measurement decreased significantly during the post-taper period (−4.7%, moderate effect, *d* = 1.0; *p* < 0.01). Total times during repeated sprints increased significantly (+1.6%, small effect, *d* = 0.4, *p* < 0.05) during post-taper. However, no significant difference was observed in sprint decrement. Post-taper, no significant changes were seen in physical and physiological measurements of SSG, except for RPE (+8.1%, large effect, *d* = 1.4, *p* < 0.01).

## 4 Discussion

The study investigated the effects of a 3-day step taper on lower-limb explosive power, sprint performance, and physical and physiological responses during small-sided games (SSGs) in professional soccer players. The present findings showed a significant decrease in training load during the taper phase compared to the corresponding day of the pre-taper microcycle after a 50% reduction in training duration while maintaining intensity. This tapering appears to be associated with a significant improvement in lower extremity power, a decrease in both peak time and total time during repeated sprints, and enhancements in total and high-speed covered distance, as well as in accelerations and decelerations during SSGs in the post-taper phase compared to pre-taper conditions.

In the current data, we observed a significant time by group interaction for the CMJAS, sprint distance covered during SSGs, and RPE after SSGs. However, no significant interaction was found for the other parameters, likely due to the short intervention duration, which is insufficient for the complete regeneration of some physiological and metabolic variables. Neuromuscular power typically rebounds faster than metabolic homeostasis; for instance, markers of muscle damage and hormonal balance often require more than 72 h to return to baseline levels following intense soccer-specific loading (22, 23). Furthermore, while a 3-day step-taper can effectively “unmask” performance by reducing acute fatigue (5, 6), it may be too brief to induce significant changes in central cardiovascular markers like maximal heart rate, which generally require longer tapering periods to manifest (8, 9). This aligns with previous research suggesting that structural muscle repair and full glycogen resynthesis are time-dependent processes that may exceed the duration of a short-term microcycle taper (24, 25). Furthermore, the primary goal of tapering is not to observe significant improvements but to prevent performance decline or loss, making it crucial to consider this evidence when interpreting results. Consequently, analyzing the data using the Student's *t*-test, effect size, and percentage differences will enable a comprehensive understanding of the findings.

Tapering effectiveness in soccer remains under investigation, and no prior study has tested a short microcycle step taper in professionals. In the present study, we implemented a taper microcycle characterized by a 50% reduction in training session durations while maintaining the initial intensity, following a 4-week mesocycle defined by a progressive training load in the TG. Consequently, this resulted in an overall reduction of 46%–49% in training load across the 3-day taper. It is well known that training intensity is a crucial factor in adaptation outcomes during tapering and should remain unchanged even with reduced training loads (8, 9). Indeed, Krespi et al. (9) found that the tapering method, which involves altering volume while maintaining intensity, is more effective in maximizing speed, power, and endurance performances in junior soccer players. In this context, lower extremity power and repeated short and intense efforts are recognized as key factors in determining success in soccer (16, 17, 20, 26), and both are related to various aspects of soccer performance that are important to monitor and assess (16, 20).

The current data shows that after the taper phase, there was a significant improvement in CMJAS (large effect; *d*: 1.5; +5.82%) and a decrease in peak time and total time (small effect; *d*: 0.3; −1.6% and −1.4%) compared to the pre-taper phase in the TG. Although these improvements in lower extremity power and repeated sprint performance appear “small” and “large”, they are consistent with previous studies that have reported improved high-intensity performance and muscle power after tapering in team sports and soccer (7–9, 23). The improvements observed after tapering could be attributed to several previously reported factors. Indeed, these factors include an increase in muscle strength and power, which has been observed after a taper period through the reduction of muscle damage, an increase in anabolism, and an increase in muscle glycogen reserves (5, 6). Certainly, increased muscle glycogen and aerobic enzyme activity are crucial for improved repeated sprint performance in professional soccer players (22, 27). While some improvements yielded “small” effect sizes, they are of high practical relevance in professional soccer, where marginal gains in explosive power and running volume can provide a meaningful competitive edge during congested match schedules (28). This is further supported by Carling et al. (10), who demonstrated that superior repeated-sprint ability is directly linked to an increased number of high-intensity actions during official professional matches, particularly those interspersed by short recovery durations. Unfortunately, the absence of invasive measurements could be considered a major limitation of this study. The players' regular exposure to these measurements within their routine training program guaranteed their comfort during evaluation, enhancing data precision. This also supported the attribution of observed performance enhancements to the effects of tapering.

Surprisingly, CG demonstrated a significant decrease in CMJAS performance (moderate effect; *d*: 1; −4.7%) and repeated sprint performance (small effect; *d*: 0.1 and 0.3 in peak and total time, approximately; −2%) during the second trial. This decline in performance is likely due to the continued training with high load, leading to the onset of fatigue. In this context, several studies have shown the negative effects of fatigue onset in the short term on this type of exercise performance (24, 25, 29). Previous research suggests that tapering can enhance oxidative enzymatic activity, potentially boosting oxygen extraction and recovery between sprints and preventing the onset of fatigue (30, 31). However, the difference between pre- and post-taper sprint decrement is not statistically significant for both groups (trivial effect; TG: *d*: 0.4; 13.13% and CG: *d*: 0.2; 14.79%). This finding aligns with earlier studies indicating the low reliability and high variability inherent in sprint decrement and fatigue indices during repeated sprints (17, 20).

SSGs significantly enhance soccer player training by promoting aerobic and anaerobic physical attributes, improving soccer-specific fitness, and influencing critical elements for match success (32). Despite their open-loop nature and variability in specific running performance measures during these exercises, SSGs serve as valuable tools for assessing the physical condition of soccer players (32, 33). The current TG data indicate that following the taper phase, there was a small increase in the total covered distance (*d*: 0.2; 2.2%). Moreover, accelerations and decelerations also showed moderate increases (*d*: 0.8; +15.94% and *d*: 0.6; +15.56%, respectively) after the taper. These changes are consistent with earlier reports of improved acceleration and intermittent running after tapering (6–9, 23). The average HR showed a small decrease after the taper in the TG data (*d*: 0.4; −2.77%), which may suggest that players in the TG experienced improved cardiovascular efficiency or enhanced recovery capacity following the taper. However, explaining this small decrease becomes challenging without previous literature, given that the average HR of the CG did not show significant differences. This highlights the need for further research to elucidate this mechanism during taper. In the same context, the current CG data indicate no significant changes observed in any of the physical measurements of SSG during the second trial. However, physiological parameters, i.e., RPE and maximal HR, increased during the second trial of CG (large effect for RPE; *d*: 1.4), which could probably be attributed to the continued training with high loads during the fifth week, resulting in the onset of fatigue. Notably, the higher baseline RPE observed in the CG, despite strictly matched external training loads, suggests a greater level of accumulated residual fatigue at the start of the experimental phase. This highlights the high sensitivity of subjective measures, as RPE was able to detect internal strain that was not yet apparent in GPS-derived metrics. In this context, Moalla et al. (2) confirmed that the length of mesocycle blocks could significantly influence performance in professional soccer players. Overall, the changes observed in SSGs for both groups should be analyzed and interpreted cautiously, as previous studies have shown that SSGs might not be as relevant for detecting training-induced improvements (34). The practical applications of this study recommend and encourage professional coaches and fitness staff to implement a short microcycle taper, characterized by a 50% reduction in session duration while maintaining training frequency and intensity. This strategy is particularly beneficial during congested match schedules, as it helps enhance players' physical performance while minimizing the risks of fatigue, injury, and performance decline.

### 4.1 Study limitations

The study acknowledges several limitations, including the small, single-team sample which may limit external generalizability to teams with different tactical periodization models. The short intervention (3 days) primarily reflects short-term recovery and immediate performance peaking, which might not capture the full kinetics of a longer tapering period. Furthermore, the lack of biochemical markers (e.g., hormonal status) and neuromuscular assessments limits a comprehensive mechanistic interpretation; specifically, it remains unclear whether the gains were driven by neuro-endocrine recovery or the mitigation of peripheral fatigue. Consequently, while these findings provide valuable practical insights, they should be interpreted with caution regarding their broader application to different competitive contexts.

### 4.2 Future perspectives

Future research should transition toward more actionable study designs. Specifically, multi-group randomized controlled trials are needed to compare different tapering magnitudes (e.g., a ∼30% vs. ∼60% reduction in training volume) to establish the optimal dose-response relationship for professional soccer players within congested schedules. Furthermore, future investigations should explore individualized tapering strategies personalized to players' physiological profiles and position-specific demands. For instance, optimizing performance peaking may require distinct approaches for wide players, who must maintain high-speed running efficiency, vs. central defenders, who may benefit more from a primary focus on neuromuscular power and accelerative/decelerative capacity. Finally, incorporating monitoring of biochemical markers (e.g., salivary cortisol and creatine kinase) alongside neuromuscular assessments would provide a definitive test of the physiological mechanisms underpinning the effectiveness of these short-term taper models in elite professional environments.

## 5 Conclusion

The present findings indicate that a 3-day step taper, characterized by a 50% reduction in training duration while preserving intensity and frequency, effectively enhances lower-limb explosive power, repeated-sprint ability, and SSG high-intensity running performance in professional soccer players. The study highlights the effectiveness of short taper protocols for improving performance and reducing fatigue, although caution is used due to the small population sample. Future research should involve larger and more diverse populations and also consider physiological markers for a more informative examination of adaptation mechanisms.

## Data Availability

The dataset supporting the results (35) of this study has been deposited in Zenodo under the DOI: https://doi.org/10.5281/zenodo.17373303. The dataset (35) includes anonymized raw values for all performance tests, RPE, and GPS metrics. Data are shared under the CC-BY 4.0 license. Additional information can be obtained from the corresponding author upon reasonable request.
